# Surgeon’s perceptions on 3D visualization methods in parotid gland tumor surgery

**DOI:** 10.3389/fonc.2025.1655175

**Published:** 2025-09-22

**Authors:** Manon C. M. Moll, Coralie Arends, Loes M. M. Braun, Matthijs H. Valstar, Ludi E. Smeele, Charlotte L. Zuur, Maarten J. A. van Alphen, Luc H. Karssemakers

**Affiliations:** ^1^ Netherlands Cancer Institute, Antoni van Leeuwenhoek, Department of Head and Neck Surgery and Oncology, Amsterdam, Netherlands; ^2^ Netherlands Cancer Institute, Antoni van Leeuwenhoek, Department of Head and Neck Surgery and Oncology – Verwelius 3D Lab, Amsterdam, Netherlands; ^3^ Netherlands Cancer Institute, Antoni van Leeuwenhoek, Department of Radiology, Amsterdam, Netherlands

**Keywords:** facial nerve, parotidectomy, segmentation, 3D printing, AR visualization

## Abstract

**Introduction:**

Recent advances in high-resolution MRI and reconstruction techniques offer new opportunities to enhance visualization of the facial nerve and its relationship to parotid tumors in 3D models. The aim of this study is twofold: first, to assess the technical feasibility of generating three-dimensional printed anatomical models from MRI data. Second, to evaluate surgeons’ perceptions of three different visualization methods (3D models on a 2D screen, 3D-printed models, and augmented reality (AR) holograms) to identify the most advantageous method for surgeons performing parotid gland tumor surgery.

**Methods:**

Fifteen surgeons (otolaryngologists, cranio-maxillofacial, and head and neck) evaluated four clinical Cases using all three visualization methods, in addition to conventional MRI. Participants completed structured questionnaires assessing anatomical clarity, clinical utility, and perceived usefulness and ease of use. Statistical analyses included Friedman and Wilcoxon signed-rank tests, as well as Spearman correlations.

**Results:**

AR holograms achieved the highest median scores for tumor visibility, while all methods performed equally on anatomical landmark visibility. Significant differences in surgical decision-making were observed across cases, with 3D visualizations influencing preferences for surgical approach and perceived risk of facial nerve injury. For intended use, screen-based 3D models and conventional MRI were rated highest for patient consultation and preoperative planning, while intraoperative use received lower scores overall. Perceived usefulness and perceived ease of use scores were highest for AR and 2D screen models. The 3D-printed models were generally rated lower, though some value was noted for patient communication.

**Conclusion:**

Printing the facial nerve in relation to a tumor is feasible, despite technical challenges, for which solutions are provided. For clinical care, an anticipated role in preoperative patient consultation and surgical planning of 3D models was favored more than intraoperative use. Among the visualization methods, 3D-printed models were perceived as less effective than those displayed on a 2D screen or in AR. 3D models can serve as valuable adjuncts, but they do not replace conventional MRI.

## 1 Introduction

Facial nerve injury remains an essential complication in parotid tumor surgery, with the potential to impact a patient’s quality of life significantly (1–3). Despite the use of anatomical landmarks and intraoperative facial nerve monitoring to identify the facial nerve, facial nerve management during surgery remains a challenge (4).

Advancements in magnetic resonance imaging (MRI), particularly high-resolution neurography sequences, now allow for improved visualization of the facial nerve and other nerves (5, 6). These imaging data can be reconstructed into three-dimensional (3D) models, providing enhanced insight into the spatial relationship between the tumor and the facial nerve. These models can aid in patient counseling, enhance preoperative planning, and support intraoperative procedures (7).

3D models can be visualized in different visualization formats. On a two-dimensional (2D) screen, users can interact with the model by rotating and zooming using a computer mouse or through touch gestures on a tablet. Alternatively, they can be translated into tactile models via 3D printing (additive manufacturing) or experienced through augmented reality (AR) using a head-mounted device. These visualization methods have been used in several surgical fields. 2D screen models have shown promise in skull base tumor surgery and neurovascular decompression surgery (8–10). Lin et al. (11) reported that patient-specific 3D-printed models were beneficial for simulating skull base tumor surgeries. In prostate cancer, where nerve preservation is crucial, both 3D-printed models and AR models have proven helpful in surgical planning and patient counseling (12–14).

In parotid gland surgery, the 3D visualization of the spatial relationship between the tumor and the facial nerve could aid in facial nerve management. Several MRI sequences have proven effective in visualizing the facial nerve and surrounding structures for 3D reconstruction (22). Saadya et al. (15) and Hu et al. (16) investigated the application of AR visualization in parotid surgery, yielding promising results. To date, no studies have examined the use of 3D-printed facial nerve models in this context. Consequently, it remains unclear which visualization method, 2D screen, AR, or 3D-printed model, is preferred by surgeons. The aim of this study is twofold. First, we want to assess the technical feasibility of creating three-dimensional printed models. Second, we evaluate the surgeon’s perception of those visualization methods to learn which technique is more advantageous for surgeons.

## 2 Materials and methods

### 2.1 Participants

This prospective study was approved by the Institutional Review Board of the Netherlands Cancer Institute – Antoni van Leeuwenhoek (IRBd24-110). Questionnaires were administered between January 2025 and May 2025 to a panel of physicians (otolaryngologists (ENT) surgeons, maxillofacial surgeons, and head and neck surgeons) who regularly perform parotid gland surgery. The physicians are recruited through a direct approach and ‘snowball sampling’. No group interaction occurred during the study. We collected data on participants’ age, type of hospital affiliation, and years of experience in parotid gland tumor surgery.

### 2.2 Data acquisition

**Figure 1:**
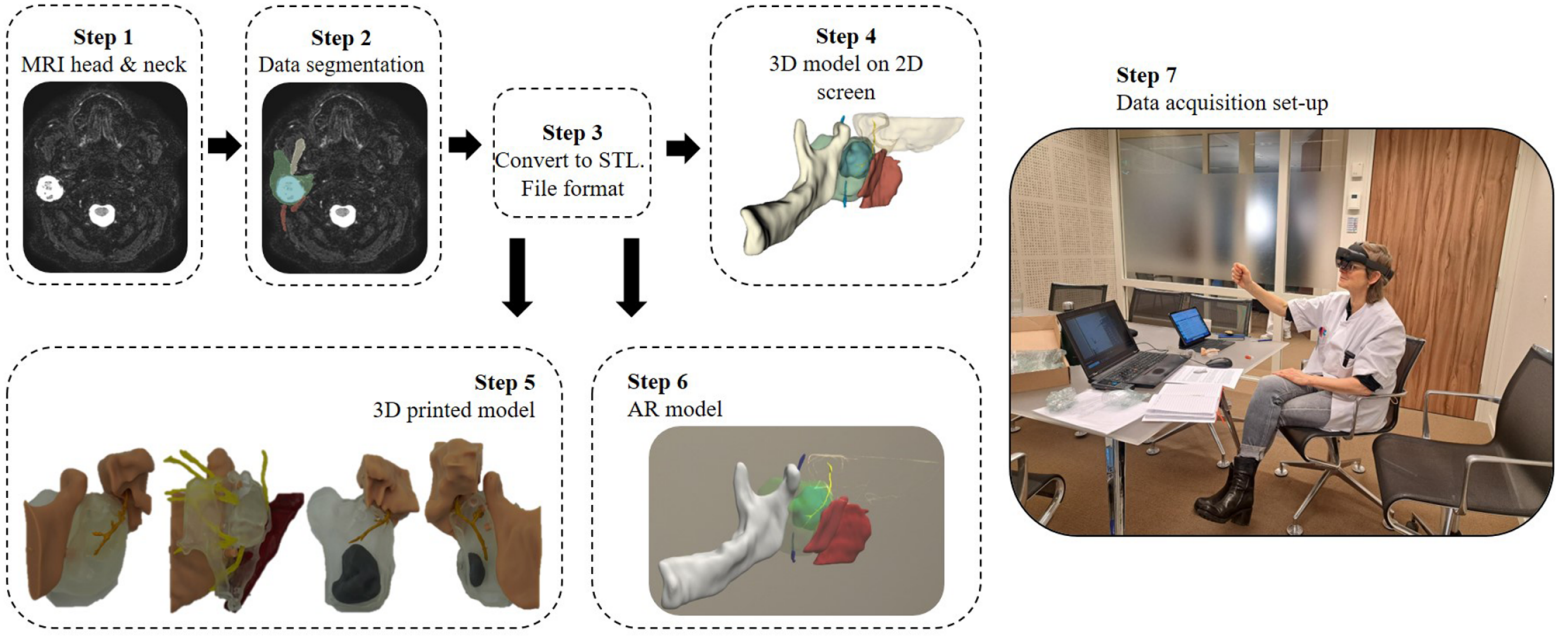
This workflow illustrates the construction process of various 3D model visualization methods related to the facial nerve, including those surrounding a parotid tumor and adjacent landmark structures, as well as the setup used during data acquisition. Patient-specific MRI data were used to segment anatomical structures, which were then converted into stereolithography (STL) files for 3D printing. These STL files served as the basis for three visualization methods: displaying the 3D model on a 2D screen, fabricating a physical 3D-printed model, and integrating it into an augmented reality (AR) environment using the HoloLens 2. Finally, during the assessment, the participating surgeon assessed the conventional MRI and three 3D model visualization methods.

The panel of surgeons was asked to independently assess four clinical Cases using four visualization methods presented in a fixed sequential order: conventional MRI images, a 3D model on a 2D screen, a 3D-printed model, and an AR hologram. An overview of the evaluation process is illustrated in **Figure 1**.

After reviewing the conventional MRI and each visualization method per Case, participants completed a questionnaire evaluating several aspects: visualization quality of the tumor and anatomical landmarks relevant to facial nerve identification, the proposed surgical approach, perceived risk of facial nerve injury, and the likelihood of using each method for patient consultation, preoperative planning, and intraoperative decision-making. For each 3D visualization method, the perceived added value compared to conventional MRI was assessed across these intended uses. The questionnaire, developed specifically for this study and not previously validated, used a 7-point Likert scale (1 = strongly disagree, 7 = strongly agree). The complete questionnaire is provided in **Supplementary Data**. One question was excluded from analysis as it contained a double negation in its phrasing.

Each physician completed the full cycle of evaluations and questionnaires for all four Cases. After completing the four Cases, participants were asked to complete an additional validated technology acceptance model (TAM) based questionnaire assessing the Perceived Usefulness (PU) and Perceived Ease of Use (PEOU) of the different 3D model visualization methods (17).

### 2.3 Case models

Four clinical Cases were selected for this study. These Cases are patients who have previously signed informed consent for the use of their clinical data in an earlier study and provided consent for future related studies, which were approved by the same Institutional Review Board (IRBd22-343). Their characteristics are summarized in **Table 1**. Two of the selected neoplasms were benign, while the other two were malignant.

**Table 1:** Summary of case characteristics.

Case number	Sex, age (years)	Side	Tumor histology and diameter
1	F, 75	R	Pleomorphic adenoma, 3.2 cm
2	M, 27	L	Adenocarcinoma, 2.6 cm
3	M, 44	R	Muco-epidermoid carcinoma, 2.5 cm
4	M, 53	L	Warthin tumor, 1.9 cm

Case 1: a pleomorphic adenoma located in the superficial lobe, in close contact with the facial nerve, displacing it medially. Case 2: an adenocarcinoma encapsulating the facial nerve. Case 3: a mucoepidermoid carcinoma situated in the inferior part of the parotid gland, closely related to the lower division of the facial nerve. Case 4: a superficial Warthin tumor with no contact with the facial nerve.

### 2.4 MRI data and 3D segmentation

MRI data were acquired using the head and neck protocol on a 3.0-T Philips dStream Achieva scanner in combination with a 32-channel receiver head coil (Philips Healthcare, Best, The Netherlands). Three series from this protocol were included in the analysis. The conventional MRI, reviewed by the participating surgeons, consisted of a T1-weighted turbo spin-echo (TSE) sequence and a T2-weighted Short Tau Inversion Recovery (STIR) sequence. Parameters for the T1 TSE sequence were: repetition time (TR) 500–900 ms, echo time (TE) 16.7 ms, reconstructed voxel size 0.46 x 0.46 x 3 mm, and TSE factor 6. T2 STIR sequence parameters were: shortest TR, TE 20 ms, reconstructed voxel size, 0.44 x 0.44 x 3 mm, and TSE factor 12.

To visualize the facial nerve, we employed the NerveVIEW sequence, a non-contrast-enhanced 3D T2-weighted turbo spin echo acquisition. This sequence uses extended echo train sweeps with variable flip angles to preserve magnetization and achieve high spatial resolution. Additionally, a motion-sensitized driven equilibrium pre-pulse, combined with STIR, effectively suppresses signals from blood vessels and fat, thereby enhancing nerve visibility as hyperintense structures. NerveVIEW sequence parameters were as follows: acquired voxel size 0.89 x 0.90 x 0.90 mm, reconstructed voxel size 0.50 x 0.50 x 0.90 mm, TR 2300 ms, TE 188 ms, STIR inversion time 250 ms, 100 slices, TSE factor 43, and field of view 200 x 200 x 90 mm. The surgeons did not review the NerveVIEW during assessment. While this neurography sequence provides visualization of cranial nerves, it gives limited information on surrounding anatomical structures. Additionally, it has a relatively small field of view compared to standard MRI sequences.

The MRI Digital Imaging and Communications in Medicine (DICOM) data were segmented and reconstructed into 3D models using the open-source medical imaging and visualization software, 3D Slicer version 5.0.3 (Surgical Planning Laboratory, Harvard University, Boston, MA, USA) (18). Image alignment was performed using landmark-based registration, with the NerveVIEW sequence as the fixed reference. The NerveVIEW sequence was utilized to visualize and segment the facial nerve. In contrast, adjacent anatomical structures (tumor, parotid gland, digastric muscle, sternocleidomastoid muscle, surgical pointer, retromandibular vein, mandible) were segmented from the T1- and T2-weighted sequences. Manual segmentation of the facial nerve and retromandibular vein was conducted using the “Paint” tool within the Segment Editor module. The remaining structures were segmented using a combination of the “Paint” and “Fill Between Slices” functions.

### 2.5 3D print process

STL files of the facial nerve, tumor, parotid gland, digastric muscle, pointer, and mandible were exported to 3-Matic software (Materialise, Leuven, Belgium) for pre-processing before 3D printing. The sternocleidomastoid muscle and retromandibular vein were excluded, as they are not primary anatomical landmarks for the facial nerve in parotid gland surgery, and including too many structures proved impractical.

To prepare the model and reduce the structure sizes, the pointer and mandible were trimmed to retain only the key anatomical landmarks relevant for facial nerve identification. These structures, along with the digastric muscle, were connected to the parotid gland using the pin-and-hole feature in 3-Matic. The parotid gland was designed as a hollow structure with an offset of 2 mm. At the facial nerve’s entry point into the gland, the parotid model was sliced in the sagittal plane, ensuring the tumor remained intact and dividing the parotid gland into two parts.

The facial nerve was fixated to the parotid gland using a small baseplate at its entry point and secured with fast-acting adhesive to ensure anatomically accurate positioning. During the fabrication process, it was observed that the printed facial nerve occasionally had a thickness of less than 1 mm, making it fragile and prone to damage. To improve durability without altering the spatial distance between the facial nerve and tumor, the nerve was selectively thickened on the side opposite the tumor. For instance, in Case 1, where the tumor was located superficially, the facial nerve was thickened on the medial side. To accommodate the nerve’s course through the mastoid and its exit via the stylomastoid foramen, a cylindrical opening was added to the mastoid, allowing separate printing and assembly. The **Supplementary Movie** demonstrates the processed components in the 3-Matic environment.

**Figure 2:**
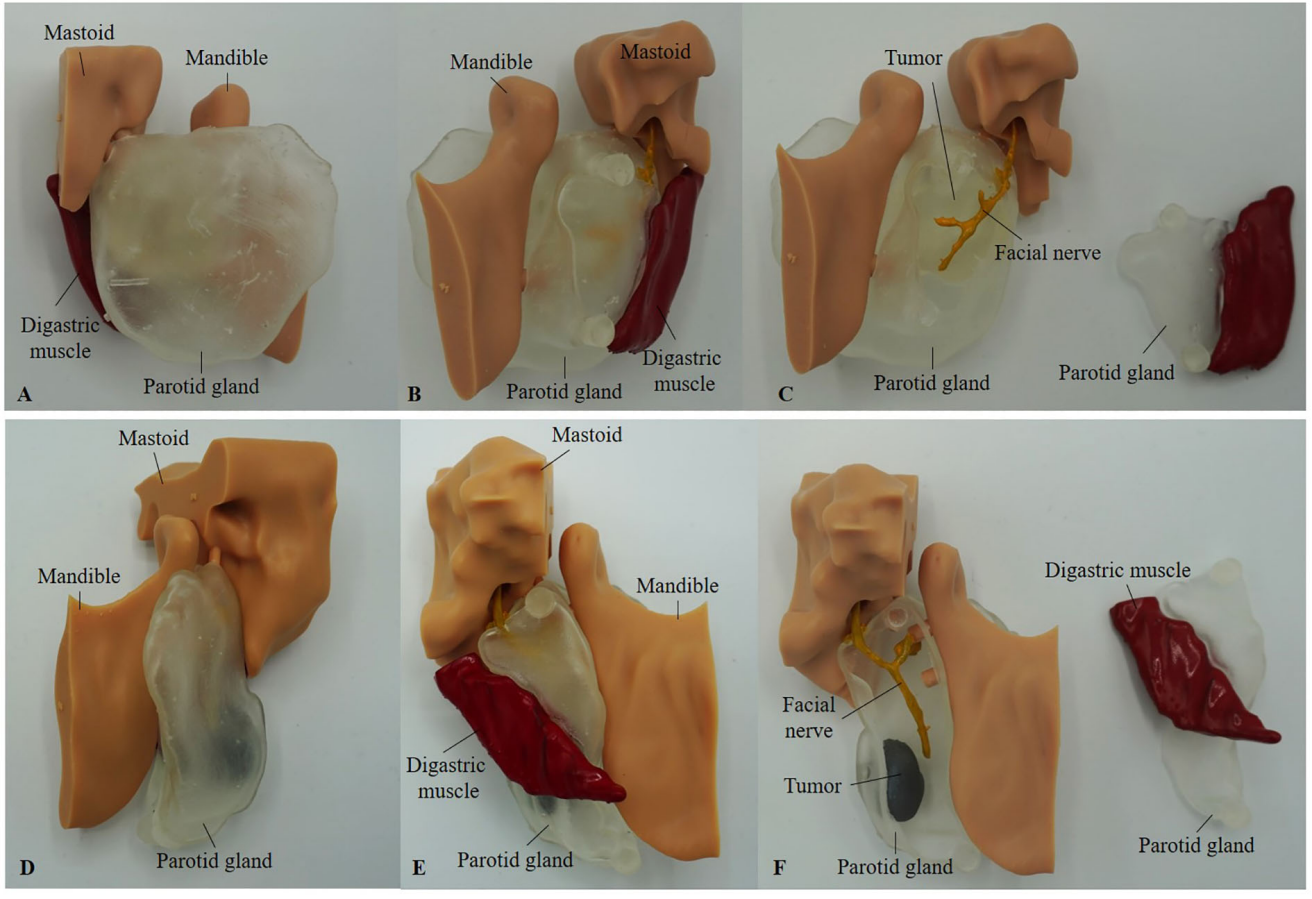
Printed models of Case 1 **(A-C)** and Case 4 **(D-F)**, shown from the lateral side **(A, D)** and medial view **(B, E)**. **(C, F)** present the medial view with the smaller parotid gland segment, including the digastric muscle, detached. In Case 1 **(C)**, the tumor was printed in clear resin due to its large size and is not colored. In contrast, in Case 4 **(F)**, the tumor was printed separately in gray resin and inserted into the parotid gland using the pin-and-hole method.

Anatomical models were printed at a 1:1 scale using the Formlabs FORM 3B printer (Formlabs, Somerville, United Kingdom) (**Figure 2**). When feasible, the tumor was printed separately in gray resin and inserted into the sagittal opening of the parotid gland using the pin-and-hole method, and fixated with adhesive. In Cases involving larger tumors (e.g., Cases 1 and 2), the tumor was printed as an integrated part of the parotid gland in clear resin to ensure structural stability and accurate spatial representation.

The facial nerve, tumor (in Cases 3 and 4), and the digastric muscle were printed using gray resin. Due to the complexity of Case 2, the parotid gland, tumor, and facial nerve were printed as one piece. The mandible and mastoid pointer were printed using standard model resin. Attempts to print using a filament-based printer were unsuccessful due to the insufficient resolution required for fine anatomical details necessary for accurate assembly. After printing, the facial nerve and digastric muscle were manually colored yellow and red, respectively, for enhanced visibility. The digastric muscle was affixed to one half of the parotid gland. The pin-and-hole connections between the mandible and mastoid components, as well as the parotid gland, allow these parts to be attached and detached as modular elements.

### 2.6 Augmented reality

AR visualization was performed using the HoloLens 2 headset (Microsoft, United States). The Lumi software platform (Augmedit, The Netherlands) was used to display the 3D STL models for each Case. Within the Lumi interface, individual anatomical structures could be toggled on or off, allowing users to focus on specific elements. Using hand gestures, the models could be rotated, zoomed, and repositioned in real-time. Additionally, the software enabled overlaying the original imaging data, allowing users to view the 3D model in alignment with the corresponding radiological scans, thereby enhancing anatomical context and spatial understanding.

### 2.7 Analysis

Data from the questionnaires were analyzed using descriptive statistics, including the median and interquartile range (IQR), for each visualization method and Case. Responses to the TAM-based questionnaire were grouped into PU and PEOU. The internal consistency of the PU and PEOU constructs was evaluated using Cronbach’s alpha. Differences between methods were assessed using the Friedman test for repeated measures. When significant differences were found, *post-hoc* pairwise comparisons were conducted using the Wilcoxon signed-rank test with Bonferroni correction. A p-value of < 0.05 was considered statistically significant. To explore relationships between Perceived Usefulness, Perceived Ease of Use, the likelihood of future clinical use, and years of experience of the participating surgeons, Spearman correlation coefficients were calculated. Statistical analyses were performed using RStudio (PBC, Boston, MA, URL http://www.rstudio.com).

Following the completion of the questionnaires, participants were asked additional questions regarding their ability to orient the 3D models, whether any anatomical structures were missing, and how the models could be improved to enhance their clinical applicability. The qualitative feedback collected was systematically reviewed and thematically analyzed to identify recurring insights and limitations associated with each visualization method. Additionally, the time and cost associated with each visualization method were analyzed to assess its practical feasibility.

## 3 Results

### 3.1 Model presentation and participant demographics

All four clinical cases were presented to the participating surgeons using three visualization formats: 3D models on a 2D screen, physical 3D-printed models, and AR models. A total of 15 surgeons participated in the study, including three females and 13 males. The mean age was 47.9 years (SD: 9.9), with a range of 31 to 61 years.

Of the participants, nine were trained as ENT specialists and six as maxillofacial surgeons. Two surgeons had not completed the head and neck fellowship program introduced in the Netherlands in 1996, while two others were in the process of completing the fellowship at the time of the study. The participants’ independent experience performing parotidectomy procedures ranged from 5 months to 27 years. Thirteen of the surgeons were affiliated with one of the head and neck centers in the Netherlands.

### 3.2 Study-specific questionnaire

Questionnaire outcomes are described in **Table 2**.

**Table 2:** Descriptive statistical outcomes, mean and interquartile range (IQR), grouped per questionnaire item for the study-specific questionnaire and technology acceptance model questionnaire, with questions on perceived usefulness and perceived ease of use.

Score: Median (IQR)					
Questionnaire item		Conventional MRI	3D model on 2D screen	Printed 3D model	AR hologram
1	Anatomy visibility: tumor	6 (6-6)	6 (6-7)	6 (5-7)	7 (6-7)
2	Anatomy visibility: landmarks for facial nerve identification	6 (4.8-6)	6 (6-7)	6 (5-7)	6 (6-7)
3	Surgical approach: partial parotidectomy	6 (2-6)	6 (2-6)	5.5 (2-6)	4.5 (2-6)
4	Surgical approach: total parotidectomy	2 (1-6)	2 (1-6)	2 (1-6)	2 (1-6)
5	Surgical approach: extracapsular dissection	2 (1-5)	2 (1-5)	2 (1-5)	2 (1-5.3)
6	Risk of facial nerve injury: upper division	3 (2-5)	3 (2-6)	3 (2-6)	3 (2-6)
7	Risk of facial nerve injury: lower division	4 (2-6)	4.5 (2-6)	4.5 (2-6.3)	5 (2-6)
8	Intended use: consultation with the patient	6 (5-6.3)	6 (5-7)	5 (4-6)	5 (3-5.3)
9	Intended use: preoperative planning	6 (6-7)	6 (6-7)	6 (4-6)	6 (5-7)
10	Intended use: intraoperative use	4 (2-5.3)	3.5 (2-5)	3 (2-4)	3 (2-4.3)
11	Added value: consultation with the patient*	–	6 (5-7)	5 (6.3-2.3)	5 (4-6)
12	Added value: preoperative planning*	–	6 (6-7)	6 (4-6.3)	6 (5.7-7)
13	Added value: intraoperative use*	–	4 (2-6)	3 (2-5)	4 (2-6)
	Perceived Usefulness	–	6 (5-7)	4 (3-5)	6 (5-7)
	Perceived Ease of Use	–	6 (6-7)	5 (4-6)	6 (5-6)

*Compared to conventional MRI.

#### 3.2.1 Anatomy visibility

**Figure 3:**
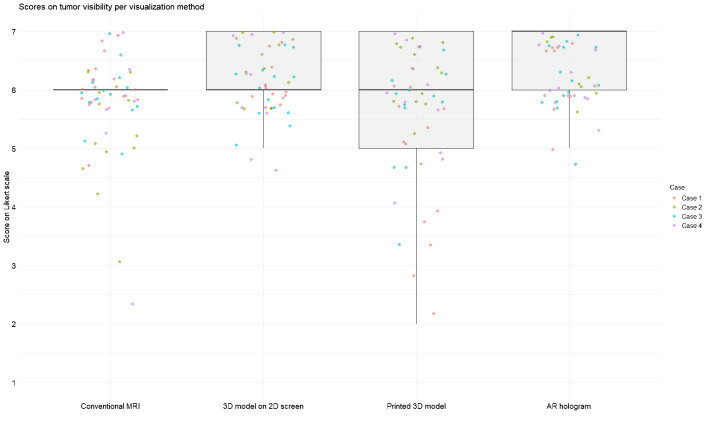
Scores for tumor visibility across different visualization methods. Each jitter represents an individual Case score. Box plots indicate the distribution of scores per modality. Scores were rated on a 7-point Likert scale (1 = strongly disagree, 7 = strongly agree).

**Figure 4:**
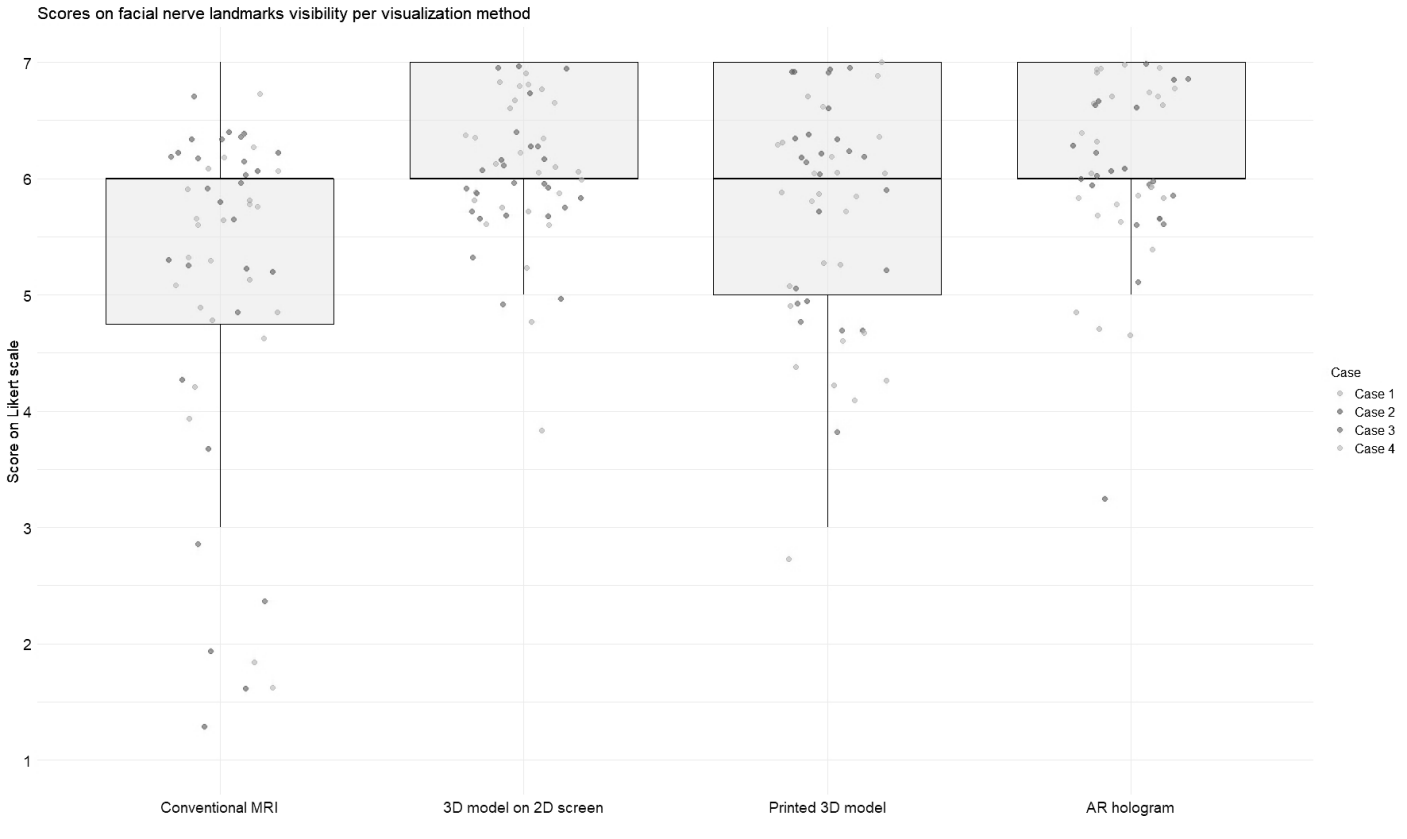
Scores for visibility of landmarks for facial nerve identification across different visualization methods. Each jitter represents an individual Case score. Box plots indicate the distribution of scores per modality. Scores were rated on a 7-point Likert scale (1 = strongly disagree, 7 = strongly agree).

**Figures 3** and **4** illustrate the distribution of anatomy visibility scores per visualization method for each Case. **Figure 3** presents scores related to tumor visibility, corresponding to the results of Question 1 in **Table 2**. The distribution of the scores on tumor visibility is larger in the conventional MRI group, especially in Case 2, as well as in the group of printed 3D models. **Figure 4** shows scores of the visibility of anatomical landmarks relevant to facial nerve identification, aligning with the outcomes of Question 2 in **Table 2**. Similar to the tumor visibility results, the conventional MRI and printed model groups show a wider distribution of scores across cases compared to the other two visualization methods.

The AR group achieved the highest median score for tumor visibility. All methods scored an equal median score on anatomical landmarks, with the most extensive distribution observed in the conventional MRI. The Friedman test revealed significant differences in modality ratings for Cases 1 (χ^2^ (3) = 19.7, p = 0.0002) and 2 (χ^2^ (3) = 24.6, p = 0.00002) in tumor visualization and in all Cases for facial nerve anatomical landmark visibility (Case 1: χ^2^(3) = 17.4, p = 0.0005; Case 2: χ^2^(3) = 18.4, p = 0.003; Case 3: χ^2^(3) = 9.3, p = 0.025; Case 4: χ^2^(3) = 8.9, p = 0.03). *Post hoc* Wilcoxon signed-rank tests indicated that for tumor visualization in Case 1, the printed 3D model scored significantly lower than the other three visualization methods. In contrast, in Case 2, conventional MRI scored significantly lower than the rest. Regarding anatomical landmark visibility, the screen-based 3D models and AR models performed significantly better in Cases 1 and 3. In contrast, in Cases 2 and 4, all three 3D methods outperformed conventional MRI.

#### 3.2.2 Surgical approach and risk of facial nerve injury

**Figure 5:**
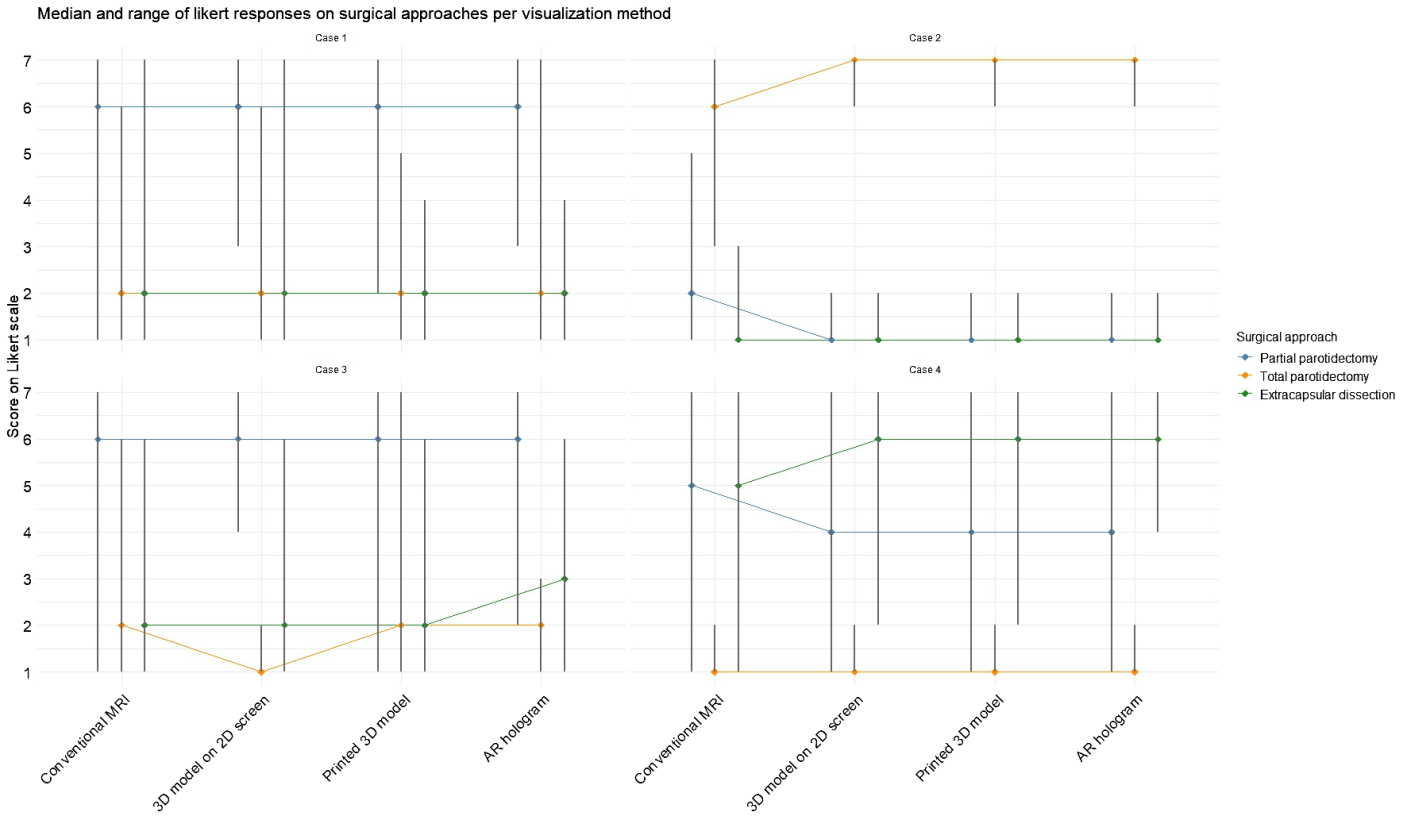
Median and range for each surgery type per case based on each visualization method. Scores were rated on a 7-point Likert scale (1 = strongly disagree, 7 = strongly agree).

In **Figure 5**, the difference in likelihood of surgical approach per case is visualized after examining each visualization method, corresponding to the results of Questions 3–5 in **Table 2**. In Cases 1 and 2, the introduction of 3D models did not alter the overall preference for a specific surgical technique. However, the range of responses narrowed for extracapsular dissection in Case 1 and across all three approaches in Case 2. In Case 3, no consistent preference emerged, whereas in Case 4, the use of 3D models led to greater consensus among participants, with a majority favoring extracapsular dissection.

The Friedman test revealed significant difference in one of the surgical decision makings with the different visualization methods: in Case 1 (χ^2^(3) = 10.4, p = 0.02) and Case 2 (χ^2^(3) = 14.0, p = 0.003) for partial parotidectomy, in Case 2 for total parotidectomy (χ^2^(3) = 19.8, p = 0.0002) and in Case 4 for extracapsular dissection (χ^2^(3) = 11.8, p = 0.008). *Post hoc* analyses revealed that conventional MRI often differed significantly from the 3D visualization methods, indicating that exposure to 3D models increased the likelihood of selecting an alternative surgical approach.

**Figure 6:**
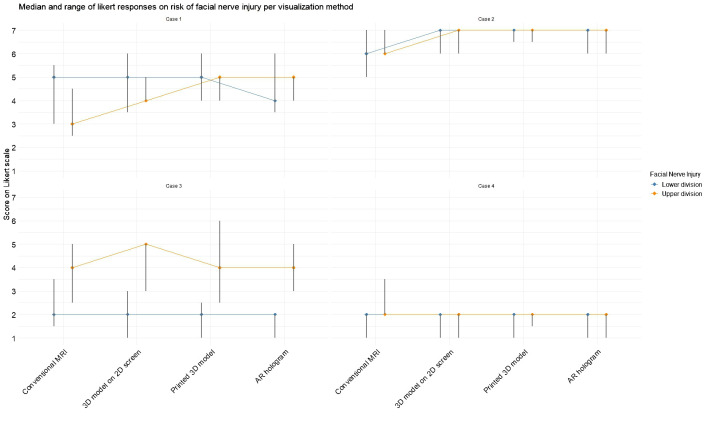
Median and range for expected facial nerve damage for the upper and lower divisions based on each visualization method. Scores were rated on a 7-point Likert scale (1 = strongly disagree, 7 = strongly agree).

**Figure 6** illustrates the changes in expected facial nerve injury across visualization methods, corresponding to the results of Questions 6 and 7 in **Table 2**. In Case 2, a greater degree of agreement was observed among participants regarding the likelihood of both lower and upper division facial nerve injuries. In Case 1, initial assessments based on conventional MRI indicated a higher probability of lower division injury. However, after viewing the AR model, expectations shifted, with upper division injury perceived as more likely than lower division injury. A statistical difference was measured with the Friedman test in expectation for upper division injury for Case 2 (χ^2^(3) = 12.9, p = 0.005) and Case 3 (χ^2^(3) = 8.6, p = 0.03), indicating a change in expectancy for nerve injury after seeing the 3D model visualizations. For the lower division, statistical significance was measured for Case 1 (χ²(3) = 14.0, p = 0.003), again indicating a change in expectancy for nerve injury after viewing the 3D model visualizations.

#### 3.2.3 Expected intended use and added value

**Figure 7:**
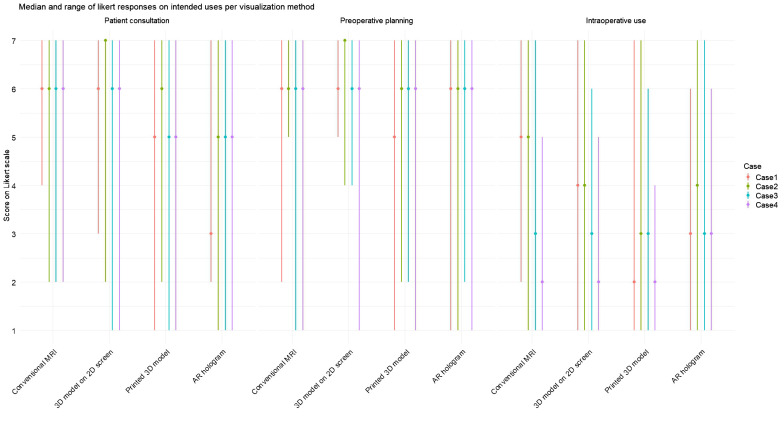
Median and range for each intended use (patient consultation, preoperative planning, and intraoperative use) on each visualization method per case. Scores were rated on a 7-point Likert scale (1 = strongly disagree, 7 = strongly agree).

**Figure 7** illustrates the median score and ranges for each intended use, corresponding to the results of Questions 8–10 in **Table 2**. For patient consultation, the highest median scores were observed for conventional MRI and the 3D model on a 2D screen. In contrast, lower scores were reported for the printed 3D model and AR hologram. For preoperative planning, all methods received a consistent median score of 6. In contrast, median scores for intraoperative use were lower across all methods, compared to the other intended uses.

Friedman tests were conducted for each expected intended use question across all visualization methods, both collectively and per case. When aggregating responses across all cases, a statistically significant effect was found only for patient consultation (χ²(3) = 16.5, p <.001). *Post-hoc* analyses revealed that conventional MRI and 3D models on screen were rated significantly higher than AR holograms and 3D-printed models.

When analyzing responses per case, statistically significant differences between visualization methods for the intended use of patient consultation were found in Case 1 (χ²(3) = 11.0, p = .01), Case 2 (χ²(3) = 24.7, p <.001), and Case 3 (χ²(3) = 12.0, p = .05). *Post-hoc* analyses confirmed that conventional MRI and 3D models on screen were rated significantly higher than AR holograms and 3D printed models. For preoperative planning, a significant difference was observed only in Case 1 (χ²(3) = 12.3, p = .007), favoring conventional MRI and 3D screen models over 3D printed models. For intraoperative use, significant differences were found in Case 1 (χ²(3) = 11.1, p = .01) and Case 2 (χ²(3) = 13.3, p = .004), again favoring MRI and 2D screen models over 3D printed models.

The data on the added value of the intended uses, as shown in Questions 11–13 of **Table 2**, revealed the highest median per modality for preoperative planning, followed by a median of 5 or 6 for usage during patient consultation. Intraoperative use was rated with the lowest median score. Statistically significant difference in clinical benefit among the three visualization methods were observed for preoperative planning in Case 1 (χ²(2) = 11.03, p = 0.004) and Case 4 χ²(2) = 8.09, p = 0.018, as well as for intraoperative use in Case 1 (χ²(2) = 6.05, p = 0.049). *Post-hoc* analysis indicated statistical inferiority of the 3D–printed model compared to screen models and AR models. For patient consultation, a significant difference was found in Case 1 (χ²(2) = 10.4, p = 0.005), with both the 3D-printed model and the 2D screen model being rated more favorably than the AR hologram.

### 3.3 Perceived usefulness and perceived ease of use

The internal consistency of PU and PEOU results per visualization methods was measured using Cronbach’s alpha coefficient. For PU, good reliability was observed for 3D on 2D screens (α = 0.89), and 3D-printed models (α = 0.98) and AR models (α = 0.97) demonstrated excellent reliability. The PEUO data demonstrated excellent reliability for all three visualization methods, with Cronbach’s alpha ranging from 0.95 to 0.98.

For PU, both the 3D model on a 2D screen and the AR hologram received a median score of 6, while the printed 3D model scored lower with a median of 4. The Friedman test revealed a statistically significant difference in PU across the three methods (χ²(2) = 11.88, p = 0.003). *Post hoc* Wilcoxon signed-rank tests showed that the 3D model on a 2D screen and the AR hologram were rated significantly higher than the 3D printed model, while no significant difference was found between the 2D screen and AR (p = 0.27).

Regarding PEOU, the 3D model on a 2D screen and AR hologram again achieved the highest median scores of 6, accompanied by narrower interquartile ranges, indicating more consistent ratings. The printed 3D model had a slightly lower median of 5 and a broader interquartile range of 2. The Friedman test revealed a significant difference (χ²(2) = 8.00, p = 0.018). Wilcoxon tests showed that the 3D model displayed on a 2D screen was rated significantly easier to use than the 3D-printed model. No significant differences were found between the 2D screen and AR or between the 3D print and AR.

Spearman correlation analyses were conducted to examine the relationship between PU and PEOU scores and the intended use for patient consultation, preoperative planning, and intraoperative use (Q8-Q10) across the three visualization methods. All visualization methods showed weak or moderate correlations between PU and PEOU scores. In the **Supplementary Tables**, the results of the Spearman correlation are described.

**Figure 8:**
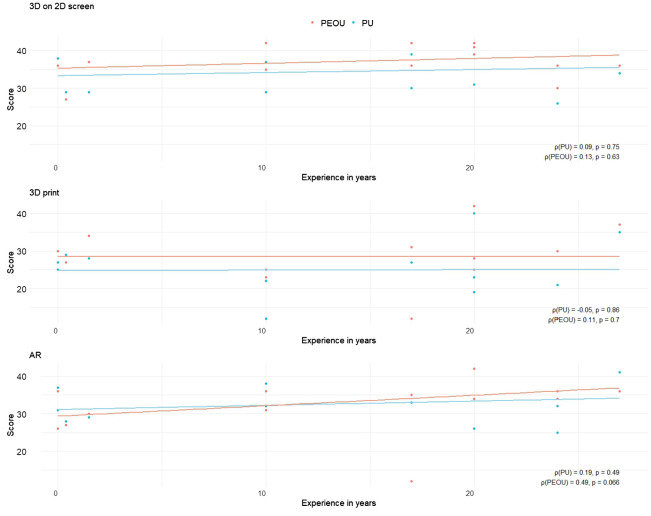
Scatter plots illustrating the relationship between participants’ years of experience and their cumulative scores on Perceived Usefulness (PU) and Perceived Ease of Use (PEOU) across different visualization methods. Each plot includes Spearman correlation coefficients (ρ) with corresponding p-values, annotated in the bottom right corner.

Additionally, Spearman correlation analyses were performed to assess the relationship between years of experience in performing parotidectomies and PU/PEUO scores for each visualization method (**Figure 8**). For both the screen-based and 3D printed models, correlations between experience and PU or PEOU were very weak. Surgeons with the same years of experience rated the PU and PEOU differently. For the AR models, the correlation between experience and PU remained very weak, but a moderately positive correlation was observed between experience and PEOU (ρ = 0.49, p = 0.066).

### 3.4 Visualization method feedback

Out of 15 participating surgeons, 14 reported being able to orient themselves adequately when viewing the models. One surgeon noted that it would be beneficial if the more distal parts of the facial nerve were also visible. A summary of qualitative feedback on the different visualization methods is presented in **Table 3**. In general, surgeons expressed a wish for the inclusion of more vascular structures, particularly in cases where the tumor was located in the deep lobe of the parotid gland. One surgeon suggested that adding a cartilage pointer could enhance the depth perception of the facial nerve in the models. Additionally, several surgeons recommended the use of artificial intelligence to accelerate the segmentation process.

**Table 3:** Summarized feedback points for all three 3D model visualization methods.

Visualization method	Feedback
3D model on 2D screen	• Use colors with better contrast to improve visual clarity. • Add left and right indicators on the screen to support faster orientation. • Increase contrast between the parotid gland and the facial nerve for better differentiation.
3D printed model	• The model should have a more professional appearance. • The current version is fragile and easily damaged. • The current cut exposes the facial nerve from a medial (inside) view, which does not accurately reflect the real surgical perspective.
AR model	• Simplify hand gesture controls to make interaction easier. • Improve contrast between the parotid gland and the facial nerve for more precise visualization.

Most surgeons preferred the 3D models on a 2D screen and the AR model. While the 3D-printed models were generally considered less favorable, some surgeons highlighted their potential value in patient consultations, suggesting that this warrants further exploration. From a logistical perspective, the 2D screen-based models were favored due to their immediate availability. The AR model was often described as a visually impressive and innovative tool that provided a clear anatomical representation with depth perception. However, surgeons also envisioned logistical challenges associated with its use. Many envisioned the HoloLens as particularly valuable for training and intraoperative guidance, especially if the models could be superimposed directly onto the patient. In the absence of such superimposition and navigation capabilities, several noted that simply using AR models in the operating room was not beneficial, as they had already internalized the 3D anatomy during preoperative planning.

### 3.5 Time and costs

The segmentation process for each model took approximately 45 minutes. An additional 45 minutes was required to prepare the segmented model for 3D printing. Loading the STL files into the HoloLens 2 for augmented reality (AR) visualization took around 5 minutes. The average material resin cost for each model was €9.70.

## 4 Discussion

Preserving the facial nerve during parotidectomy is of high importance. The introduction of high-resolution MRI neurography has enabled the detailed visualization of the facial nerve in relation to parotid tumors, offering new opportunities to enhance patient consultation, preoperative planning, and intraoperative guidance. Previous studies, including those by Hu et al. (16) and Saadya et al. (15), have demonstrated the feasibility of visualizing the facial nerve using 3D models on 2D screens and through augmented reality head-mounted displays. To our knowledge, this study is the first to present 3D-printed models of the facial nerve specifically for parotid surgery. In addition to assessing the feasibility of producing these models, we addressed a key knowledge gap by evaluating surgeons’ perceptions and preferences across different visualization methods.

Our findings suggest that 3D models offer added value to conventional MRI by visualizing the relationship between the facial nerve and the tumor, as well as its surrounding anatomy. Particularly in preoperative planning and patient consultation, 3D models have added value, with models displayed on screen and AR models being preferred over 3D-printed models. Anatomical details were most visible on the augmented reality model, providing surgeons with a more in-depth perspective. While the models did not seem to alter surgical strategies drastically, they appeared to reduce variability in planning, suggesting increased consensus among surgeons regarding operative approaches.

For patient consultation and preoperative planning, as intended use, surgeons favored either conventional MRI or the 3D model on a 2D screen. In contrast, intraoperative use of 3D models was rated lower than conventional MRI. *Post-hoc* comparisons revealed no statistically significant differences between conventional MRI and the 2D screen model, suggesting that 3D models are either equally or less likely to be consulted during surgery. Taken together with our findings on perceived added value, this indicates that conventional MRI is necessary and that 3D models can serve as valuable adjuncts, primarily because they include facial nerve information not visible on conventional MRI alone.

When evaluating PU and PEOU, the 3D-printed models scored lower than both the screen-based and AR models. Although Spearman correlation analysis did not reveal a strong relationship between PU and PEOU, qualitative feedback from surgeons suggested that ease of access and usability significantly influenced their preferences. The HoloLens was favored for preoperative planning when readily available, but concerns about setup time and integration into routine practice limited its perceived practicality. In contrast, the 2D screen model was praised for its accessibility, ease of demonstration, and environmental sustainability. Surgeons frequently noted that the printed models felt less professional and were prone to damage.

The comparative analysis across the four clinical cases revealed several patterns in how visualization methods influenced surgeons’ perceptions and decision-making. In Case 1, where the tumor is in the vicinity of the facial nerve, displacing it to the medial side, the AR model provided spatial information on the tumor-nerve relation. Consequently, the likelihood of an extracapsular dissection decreased. In Case 2, where the tumor encapsulated the facial nerve, the use of 3D visualizations led to a narrower range of responses for total parotidectomy and a more consistent assessment of facial nerve injury risk, indicating improved consensus among participants. In Case 4, where the tumor is located in the inferior part of the parotid gland, the introduction of 3D model information led to greater agreement, favoring extracapsular dissection as the surgical approach. The case-specific results highlight the value of 3D visualization methods, which depend on anatomical complexity and clinical context.

To the best of our knowledge, this is the first study to explore and compare surgeons’ perceptions of different visualization techniques, including printed 3D models, in parotid gland surgery. Earlier, Saadya et al. (15) reported positive experiences with 3D models displayed on a screen for preoperative planning and patient consultations and expressed interest in the future application of AR models. They noted challenges in accurately 3D printing the facial nerve and suggested that AR models offer superior visualization and interactivity. However, they also observed that the cost of AR models was comparable to that of 3D-printed models. In our experience, the pre-processing and post-processing required for 3D printing were time-consuming and resulted in higher labor costs. Outsourcing to specialized printing services may increase expenses further. A comprehensive cost comparison of the different visualization methods would require consideration of multiple factors, which is beyond the scope of this paper.

Hu et al. (16) used screen-based 3D models for preoperative planning and implemented AR models intraoperatively through a registration setup. A key limitation they identified was the need for surgeons to shift their visual focus between the AR display and the surgical field. They did not report the associated costs of their models.

Across surgical disciplines, comparative studies on visualization methods remain limited. Wellens et al. (19) demonstrated that converting conventional imaging to 3D models enhanced the anatomical understanding of surgeons during the preoperative planning of Wilms tumors. However, no significant differences were observed between 3D-printed and AR models. In another study assessing the impact of using 3D visualization methods for patient consultation in renal and prostate cancer, the printed models performed better than MRI imaging, screen-based models, and AR models (20). The life-sized printed models significantly improved patients’ comprehension of disease, tumor location, treatment options, and overall comfort with the proposed care. Conversely, a study comparing virtual reality (VR) and 3D-printed models for learning cardiac anatomy and pathophysiology among healthcare professionals found that VR models were preferred (21).

This study is limited by the pathology and tumor location of the four selected cases. Given the wide histological diversity of parotid tumors, we included only four cases to avoid overburdening the participating surgeons. As a result, the findings may not be generalizable across all tumor types or anatomical variations. Future research should explore which specific indications or tumor characteristics benefit most from 3D visualization. The sequence in which cases were presented to surgeons was determined by logistical considerations to optimize the use of their time. However, this approach may have introduced sequential bias, as surgeons may have gained familiarity or insight with each successive case.

Printing the facial nerve in relation to a tumor is feasible, despite technical challenges, for which solutions are provided. Surgeons perceived better visualization of the relationship between the facial nerve and parotid tumor in the 3D model, compared to conventional MRI. For clinical care, an anticipated role in preoperative patient consultation and surgical planning of 3D models was favored more than intraoperative use. Among the visualization methods, 3D-printed models were perceived as less effective than those displayed on a 2D screen or in AR. This perception may be partly attributed to the fact that participants were informed that the facial nerve had been manually thickened to enable successful printing. As a result, the printed representation may have been perceived as less anatomically accurate than the digital models, which were based directly on the original imaging data.

In our print design, we opted for pin-and-hole connections to allow the various components to be attached and detached as modular elements. This approach provided us with the flexibility to use multiple materials and apply color to individual parts using a resin printer. It also enables surgeons to customize the model according to their needs. However, this modularity makes the model more susceptible to damage. Alternatively, other print materials can be used, or the model can be printed as a single piece with multiple colors. When we tested this using our filament printer, we found that the fine details exceeded the capabilities of our hardware.

Initially, we hypothesized that less experienced surgeons might rate the PU of 3D models higher than more experienced surgeons. However, this trend was not observed in the data, indicating minimal influence of surgical experience on perceived value and usability of these modalities. Regarding the PEOU, we expected that less experienced surgeons, often younger and potentially more familiar with emerging technologies, were more comfortable with the technological innovation, especially with the HoloLens device. Contrary to this hypothesis, we found a moderate correlation between ease of use and years of experience for the AR system, indicating that more experienced surgeons found the AR system easier to use. Despite the fact that most participants had no prior experience with augmented reality device, all were able to use the HoloLens within minutes. It is possible that the PEOU scores are higher due to the new and innovative appeal rather than its actual usability (novelty or innovation bias). This interpretation is supported by several surgeons’ logistical concerns, which suggest that initial enthusiasm may not fully reflect long-term usability.

We are the first to describe the use of printed 3D models for parotid gland tumor surgery, including the facial nerve. Although the printing process presents challenges, particularly in capturing the intricate anatomy of the facial nerve and surrounding structures, we developed solutions to address these limitations. All three 3D visualization methods provided spatial information about the relationship between the tumor and the facial nerve, offering added value beyond conventional MRI, especially for preoperative patient consultations and surgical planning. However, we found no statistical differences in intended use for patient consultation, surgical planning, and intraoperative usage between the conventional MRI and 3D models methods. This suggests that the 3D models can serve as a valuable tool, but not replace conventional MRI. Among the 3D visualization methods, screen-based and AR models were perceived as more useful and user-friendly compared to 3D-printed models.

## Data Availability

The original contributions presented in the study are included in the article/**Supplementary Material**. Further inquiries can be directed to the corresponding author.
