# Supplementary material for: Surgeon’s perceptions on 3D visualization methods in parotid gland tumor surgery
Source: Front Oncol. 2025 Sep 22;15:1655175. doi: 10.3389/fonc.2025.1655175 (PMC12497603; doi:10.3389/fonc.2025.1655175)
Supplement: Supplementary file 1 [file DataSheet1.docx]

Supplementary Material

# Supplementary Data 1: Study specific questionnaire

- Visualization methods were always presented in fixed sequential order: conventional MRI images, a 3D model on a 2D screen, a 3D-printed model, and an AR model.
- Answers on 7-point Likert scale on level of agreement: 1 = Strongly disagree, 2 = Disagree, 3 = Partially Disagree, 4 = Neutral, 5 = Partially Agree, 6 = Agree, 7 = Strongly Agree
- Questions 11-13 were only asked when a 3D modality was assessed.

1. The tumor(s) can be well assessed from [visualization method].

2. The anatomic landmarks for the identification of the facial nerve can be well assessed from [visualization method].

3. Based on [visualization method], I will perform a partial parotidectomy.

4. Based on [visualization method], I will perform a total parotidectomy.

5. Based on [visualization method], I will perform an extracapsular dissection.

X. I do not expect any complications based on [visualization method].*

6. Based on this [visualization method], neuropraxia of the upper division of the facial nerve is likely.

7. Based on this [visualization method], neuropraxia of the lower division of the facial nerve is likely.

8. I expect to consult this [visualization method] during the consultation with the patient.

9. I expect to consult this [visualization method] during preoperative planning.

10. I expect to consult this [visualization method] during surgery.

11. This [visualization method] has added value compared to conventional MRI images during patient consultation.

12. This [visualization method] has added value compared to conventional MRI images during preoperative planning.

13. This [visualization method] has added value compared to conventional MRI images during intraoperative handling.

**This question was excluded for analysis due to a double negation in its phrasing, which may have led to misinterpretation.*

# Supplementary Figures and Tables

Spearman’s rank correlation coefficients between Perceived Usefulness and Perceived Ease Of Use scores of each intended use question (Q8-Q10 of the questionnaire; scores per case are averaged) for each visualization modality.

|  | Intended use | | | | | | | | |
| --- | --- | --- | --- | --- | --- | --- | --- | --- | --- |
|  | Patient consultation | | | Preoperative planning | | | Intraoperative use | | |
|  | Screen | Print | AR | Screen | Print | AR | Screen | Print | AR |
| PU | 0.4 | 0.5 | 0.0 | 0.4 | 0.4 | 0.3 | -0.04 | 0.2 | 0.5 |
| PEOU | 0.5 | 0.3 | 0.0 | 0.2 | 0.3 | 0.4 | -0.02 | 0.1 | 0.4 |
